# Impact of environmental noise exposure as an inducing factor on the prognosis of sudden sensorineural hearing loss: a retrospective case–control study

**DOI:** 10.3389/fnins.2023.1210291

**Published:** 2023-06-29

**Authors:** Yingjun Wang, Wenping Xiong, Xiao Sun, Kunpeng Lu, Fujia Duan, Haibo Wang, Mingming Wang

**Affiliations:** ^1^Department of Otolaryngology-Head and Neck Surgery, Shandong Provincial ENT Hospital, Shandong University, Jinan, China; ^2^Department of Otology Medicine, Shandong Provincial ENT Hospital, Shandong University, Jinan, China; ^3^Department of Otology Center, Shandong Provincial ENT Hospital, Shandong University, Jinan, China

**Keywords:** sudden sensorineural hearing loss, hearing loss, environmental noise, noise exposure, prognosis

## Abstract

**Objective:**

The study aimed to evaluate the clinical characteristics and prognostic factors associated with unilateral sudden sensorineural hearing loss (SSNHL) related to environmental noise exposure before its onset.

**Methods:**

A total of 50 unilateral SSNHL patients exposed to environmental noise before onset (case group) and 924 unilateral SSNHL patients without any exposure to obvious inducing factors before onset (control group) were enrolled between January 2018 and October 2022. We retrospectively analyzed differences between both groups using the chi-square test, Fisher's exact tests, independent *t*-tests, and Mann–Whitney U-tests as appropriate before and after propensity score matching (PSM) based on sex, age, and initial pure-tone average (PTA). Prognostic factors for the case group were analyzed using univariate and multivariate logistic analyses between the effective and ineffective groups.

**Results:**

Before PSM, significant differences were noted in age, sex, time to treatment, the proportion of combined diabetes mellitus, initial PTA, hearing gain, the incidence of vertigo or aural fulness, the rate of vestibular dysfunction or inner ear MRI abnormalities, the effective rate, the glucose and homocysteine levels, and the proportion of audiogram curve types (*P* < 0.05) between both groups. After PSM, compared to the control group, a longer time to treatment (Z= −3.02, *P* < 0.05), higher final PTA (Z= −2.39, *P* < 0.05), lower hearing gain (Z= −3.46, *P* < 0.05), lower rate of vestibular dysfunction (χ^2^ = 55.1, *P* < 0.001), and lower effective rate (χ^2^ = 4.87, *P* < 0.05) were observed in the case group. There was a significant difference between the audiogram curve types in both groups (χ^2^ = 14.9, *P* < 0.05). Time to treatment (95% confidence interval: 0.692–0.965, *P* < 0.05) and final PTA (95% confidence interval: 0.921–0.998, *P* < 0.05) were associated with the clinical outcomes for the case group.

**Conclusion:**

Unilateral SSNHL patients exposed to environmental noise triggers before onset showed a poorer effective rate and a lower rate of vestibular dysfunction than those who were not. The time to treatment and final PTA were associated with the prognosis of these patients.

## 1. Introduction

Sudden sensorineural hearing loss (SSNHL) is defined as sudden hearing loss (HL), unilateral or bilateral, of at least 30 dB HL across three sequential frequencies within 72 h (Chandrasekhar et al., 2019). The estimated prevalence rate of SSNHL is 5–30 per 1,00,000 people per year (Schreiber et al., 2010); a study from Germany reported that the annual incidence of SSNHL was as high as 160 per 1,00,000 people per year (Klemm et al., 2009). Recently, SSNHL has gained attention due to its increasing incidence and prolonged negative impact on patients' lives (Härkönen et al., 2017). The exact etiology of SSNHL remains unclear, but recognized etiopathogenesis processes include microcirculatory disorders, viral infection, and autoimmune or inflammatory states (Koçak et al., 2017; Li et al., 2018; Chen et al., 2019). Moreover, the factors contributing toward SSNHL development include exposure to environmental noise, sleep dysfunction, and emotional disorders although most patients presented without obvious inducing factors before the onset of SSNHL. Previous studies have assessed the relationship between sleep dysfunction, mental disorders, and SSNHL (Kim et al., 2018, 2020; Yeo et al., 2022); however, literature reporting exposure to environmental noise as a predisposing factor before the onset of SSNHL is scarce. Therefore, in this study, we retrospectively review the clinical characteristics and laboratory data of patients with unilateral SSNHL with a history of exposure to environmental noise. This study aimed to investigate the clinical characteristics, treatment outcomes, and prognostic factors of patients with SSNHL. This research may help to establish noise stimulation as a risk factor for the onset of SSNHL among patients; therefore, delay in treatment and, subsequently, compromised treatment outcomes may be avoided.

## 2. Materials and methods

### 2.1. Design/setting

The medical records of hospitalized patients diagnosed with unilateral SSNHL between January 2018 and October 2022 at our tertiary referral center were reviewed retrospectively. This study followed the STROBE guidelines.

### 2.2. Participants

The inclusion criteria for the case group were as follows: patients (1) should meet the US diagnostic criteria for SSNHL (Chandrasekhar et al., 2019); (2) with unilateral ear involvement; (3) with exposure to environmental noise triggers before onset; (4) with age between 18 and 70 years; (5) with duration time < 30 days; (6) with complete data available for audiology, vestibular function test, and MRI of the inner ear; and (7) with the length of hospital stay >7 days.

The environmental noise incorporated in this study is caused by a variety of sources, such as musical or recreational venues, machinery activity, and community noise. The exclusion criteria for the case group were as follows: patients with (1) co-existing autoimmune disease; (2) congenital or fluctuating deafness; (3) Meniere's disease; (4) middle ear malformation, otitis media, or history of middle ear surgery; (5) craniosynostosis or genetic or physical trauma-induced deafness; (6) involvement of bilateral ears; (7) occupying lesions of the pontocerebellar horn; (8) a previous history of hearing loss; (9) pre-onset with other triggers, such as sleep dysfunction, emotional stress, and cold; and (10) a history of ototoxic drug use.

For the control group, the following were the inclusion criteria: patients (1) should meet the US diagnostic criteria for SSNHL (Chandrasekhar et al., 2019); (2) with unilateral ear involvement; (3) with no obvious inducing factors before onset; (4) aged between 18 and 70 years; (5) with duration time < 30 days; (6) with complete audiology, vestibular function test, and MRI of the inner ear data; and (7) the length of hospital stay >7 days. The exclusion criteria were as follows: patients with (1) combined autoimmune disease; (2) congenital or fluctuating deafness; (3) Meniere's disease; (4) middle ear malformation, otitis media, or history of middle ear surgery; (5) craniosynostosis or genetic or physical trauma-induced deafness; (6) involvement of bilateral ears; (7) occupying lesions of the pontocerebellar horn; (8) a previous history of hearing loss or contralateral deafness; and (9) a history of ototoxic drug use.

### 2.3. Collection of data regarding medical history

Data regarding patient histories were collected for time to treatment, sex, deafness side, concomitant symptoms, such as tinnitus, vertigo, and aural fullness, and underlying diseases, such as hypertension and diabetes mellitus.

### 2.4. Audiological testing

Audiological tests performed to exclude related hearing lesions included pure-tone audiometry (GSI-61, USA), tympanometry (GSI Tympstar, USA), auditory brainstem response (ABR; IHS Smart EP, USA), and distortion product otoacoustic emission tests (DPOAE, IHS Smart EP, USA).

The audiogram curve types were classified as follows: ascending (HL limited to frequencies ≤ 1.0 kHz), sloping (HL ≥2.0 kHz), flat (HL at all frequencies with the average threshold not exceeding 80 dB HL), or total deafness (HL at all frequencies and ≥80 dB HL). The mean hearing threshold at 500 Hz, 1 kHz, 2 kHz, and 4 kHz was used to calculate the pure-tone average (PTA). The degrees of deafness were classified as mild (26–40 dB HL), moderate (41–60 dB HL), severe (61–80 dB HL), or profound (≥81 dB HL) based on the PTA. The initial PTA was measured before the entry into the study; the final PTA was measured 2 to 4 weeks after treatment. The hearing gain was defined as the difference between the initial and final PTA. Patients with a hearing gain of ≥15 dB were classified as the effective group and those with a hearing gain of < 15 dB were classified as the ineffective group (Stachler et al., 2012).

### 2.5. Vestibular function tests

To assess vestibular function, the caloric test (Ulmer VNG, v. 1.4; SYNAPSYS; Marseille, France), vestibular-evoked myogenic potentials test (Neurosoft LTD; Ivanov, Russia), video head impulse test (Ulmer, Synapsys; Marseille, France), and vestibular autorotation test (Western Systems Research; Pasadena, CA, USA) were performed. Vestibular function was considered to be impaired if the results for any of the aforementioned tests were abnormal.

### 2.6. Imaging examinations and laboratory tests

Three-dimensional (3D) fluid-attenuated inversion recovery (FLAIR) and contrast-enhanced magnetic resonance imaging (MRI) (PHILIPS, Intera, Netherlands) were performed to exclude lesions and evaluate the inner ear images. 3D T1-weighted, 3D T2-weighted, and 3D T2 FLAIR images were acquired. Inner ear images were categorized into four types: normal, increased protein content, inner ear hemorrhage, and blood–labyrinth barrier. Any of the latter three types were considered positive for an inner ear MRI abnormality.

Blood samples were collected from all admitted patients on the morning of their second day in the hospital. Blood indicators were monitored through routine blood tests, analysis of the fibrinogen level, and tests for biochemical indicators, such as the levels of total bilirubin (TB), indirect bilirubin (IB), superoxide dismutase (SOD), glucose (Glu), triglyceride (TG), cholesterol (CH), high-density lipoprotein (HDL), low-density lipoprotein (LDL), and homocysteine (Hcy). The ratios of neutrophils to lymphocytes (NLR), monocytes to lymphocytes (MLR), and platelets to lymphocytes (PLR) are defined as the ratios of neutrophils, monocytes, and platelets to lymphocytes, respectively.

### 2.7. Treatment process

During hospitalization, all patients received the following combination therapy: *Ginkgo biloba* extract (to improve blood microcirculation), dexamethasone or methylprednisolone sodium succinate (glucocorticoid), batroxobin (to lower the fibrinogen level), and methylcobalamin or mouse nerve growth factor (neurotrophic drug). Dexamethasone was administered intravenously (10 mg/day for 3 days, followed by 5 mg/day for 4 days) when the patient did not have concomitant hypertension or diabetes. Methylprednisolone sodium succinate was administered through an intravenous drip (80 mg/day for 3 days, followed by 40 mg/day for 4 days) when the patient's blood pressure and Glu levels were well-controlled. Thereafter, methylprednisolone sodium succinate post-otic injection was administered and injected continuously (40 mg/day, once every 2 days). Patients were discharged when there was no improvement after two or three consecutive pure-tone audiometry tests (twice in 1 week). Subsequently, the patients were prescribed oral *Ginkgo biloba* extract tablets and methylcobalamin.

### 2.8. Statistical analysis

Statistical analyses were performed using the SPSS 26.0 statistical package (IBM; Armonk, NY, USA). A 1:4 nearest neighbor matching was performed for sex, age, and initial PTA between the case and control groups using propensity score matching (PSM), and the caliper was set at 0.1. Chi-square and Fisher's exact tests were used to compare the categorical variables, such as sex, involvement side, hypertension, diabetes mellitus, vertigo, tinnitus, aural fullness, vestibular dysfunction, inner ear MRI abnormality, and the effective rate. Normality tests were performed mainly using the Kolmogorov–Smirnov (K-S) and Shapiro–Wilk (S-W) tests. Quantitative variables are described as the mean ± standard deviation or quartiles. Independent *t*-tests and Mann–Whitney *U* tests were used to analyze normally and non-normally distributed variables, respectively. Univariate and multivariate binary logistic regression analyses were used to assess the influence of various clinical variables on the prognosis of the case group. Differences with *P*-values < 0.05 were considered statistically significant.

## 3. Results

### 3.1. Participant characteristics

**Figure 1:**
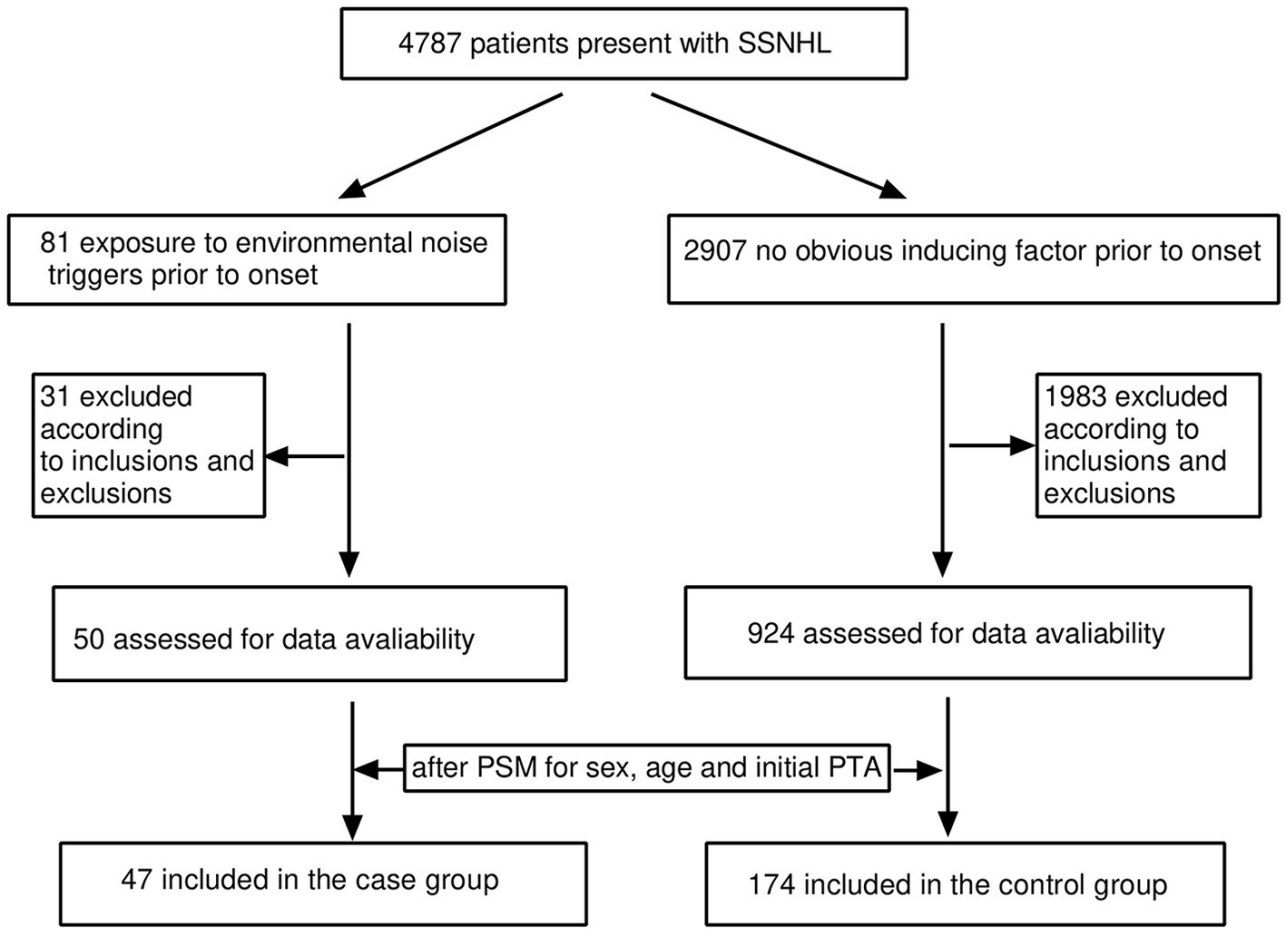
Flow diagram for screening the patients included in this study. SSNHL, sudden sensorineural hearing loss; PSM, propensity score matching; PTA, pure-tone average.

**Figure 2:**
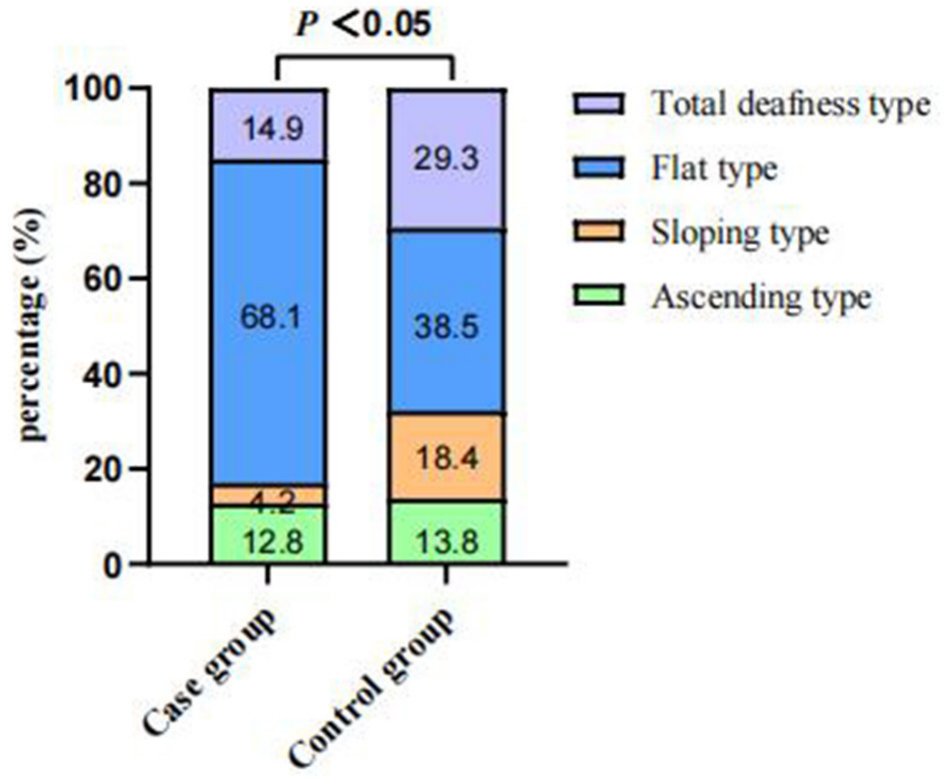
Comparisons of audiogram curve types between the case and control groups before PSM. There was a significant difference between the audiogram curve types in both groups (*P* < 0.001). PSM: propensity score matching.

**Figure 3:**
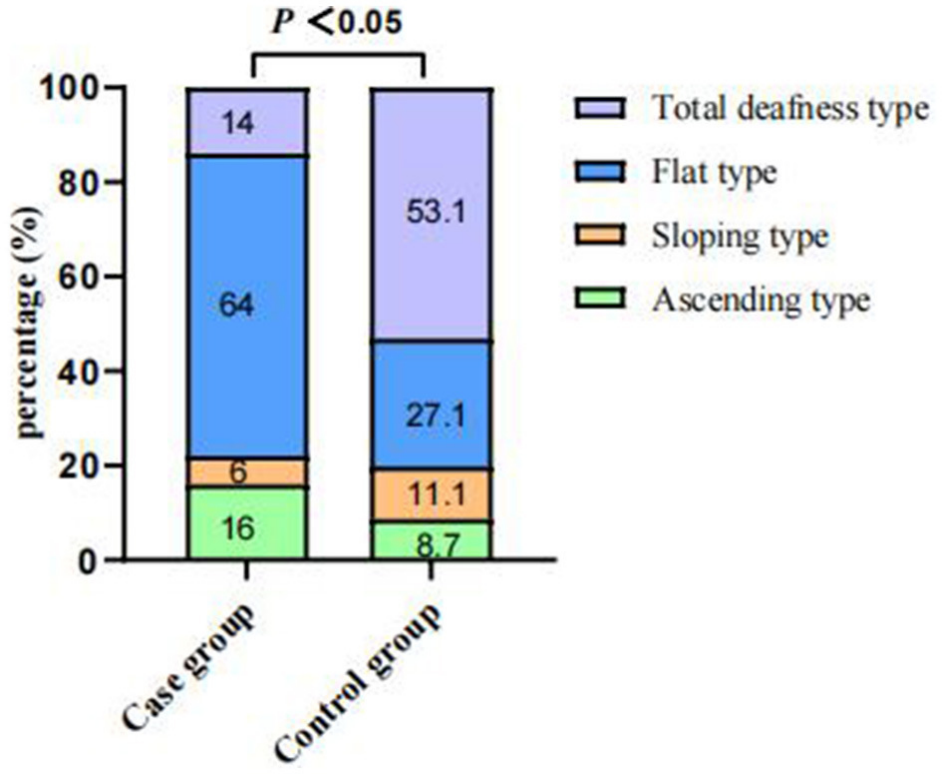
Comparisons of audiogram curve types between the case and control groups after PSM. There was a significant difference between audiogram curve types in both groups (χ^2^ = 14.9, *P* < 0.05). PSM: propensity score matching.

Of the 4,787 screened clinical records, 81 patients were exposed to environmental noise triggers before onset and 2,907 patients showed no obvious inducing factors before onset. Finally, 50 patients were enrolled in the case group and 924 patients were selected for the control group, based on the screening criteria. After PSM for sex, age, and initial PTA, 47 and 174 patients were enrolled in the case and control groups, respectively, as shown in Figure 1. Before PSM, there was a significant difference in age, sex, time to treatment, the proportion of combined diabetes mellitus, initial PTA, hearing gain, the incidence of vertigo or aural fulness, the rate of vestibular dysfunction or inner ear MRI abnormalities, the effective rate, Glu and Hcy levels, and the proportion of audiogram curve types (*P* < 0.05), as shown in Table 1 and Figure 2. After PSM, there was a significant difference in time to treatment, final PTA, hearing gain, the rate of vestibular dysfunction, and the effective rate (*P* < 0.05). The time to treatment and final PTA in the case group were larger than those in the control group (Z= −3.02 and −2.39, respectively, both *P* < 0.05). Hearing gain, the rate of vestibular dysfunction, and the effective rate in the case group were lower than those in the control group (Z= −3.46, *P* < 0.05; χ^2^= 55.1, *P* < 0.001; χ^2^= 4.87, *P* < 0.05), as shown in Table 2. After PSM, there was a significant difference between the audiogram curve types in the case and control groups (χ^2^= 14.9, *P* < 0.05), as shown in Figure 3.

**Table 1:** Demographics and clinical variables of patients (before PSM).

**Variables**	**Case group (*n* = 50)**	**Control group (*n* = 924)**	**Statistics**	***P*-value**
Age of onset	36 (30.0, 49.3)	48 (37, 56)	−3.28	0.001^*^
Males: Females	37:13	465:459	10.65	0.001^*^
Side (Left: Right)	24:26	475:449	0.22	0.64
Time to treatment (days)	10 (4, 20)	7 (3, 13)	−2.63	0.009 ^*^
Hypertension	5 (10%)	176 (19%)	2.57	0.11
Diabetes mellitus	1 (2%)	120 (12.9%)	5.26	0.02^*^
Initial PTA (dB)	58.9 ± 3.4	81.3 (56.3, 100)	−4.65	0.00^*^
Final PTA (dB)	48.3 ± 3.7	52.5 (30, 75)	−1.29	0.19
Hearing gain (dB)	6.25 (1.3, 19.1)	20 (6.3, 38.8)	−4.48	0.00^*^
Tinnitus	45 (90%)	857 (92.7%)	0.52	0.41
Vertigo	16 (32%)	513 (55.5%)	10.57	0.001^*^
Aural fulness	39 (78%)	595 (64.4%)	3.86	0.04^*^
Vestibular dysfunction	35 (70%)	768 (83.1%)	5.64	0.02^*^
Inner ear MRI (+)	12 (24%)	482 (52.2%)	15.1	0.00^*^
Thyroid dysfunction	8 (16%)	241 (26.1%)	2.53	0.11
Effective cases	15 (30%)	542 (58.7%)	15.9	0.00^*^
**Blood index**				
NLR	1.7 (1.4, 2.5)	1.8 (1.4, 2.5)	−0.38	0.70
MLR	0.19 (0.15, 0.29)	0.19 (0.14, 0.24)	−1.51	0.13
PLR	114.3 (87.9, 135.4)	115.4 (91.4, 148.4)	−0.44	0.66
TBIL (μmol/L)	13.1 (10.7, 16.5)	13.4 (10.5, 17.3)	−0.06	0.95
IBIL (μmol/L)	9.4 (7.5, 11.3)	9.8 (7.4, 12.8)	−0.46	0.64
SOD (U/mL)	158.5 (144.0, 189.5)	164 (139.3, 192)	−0.04	0.97
Fibrinogen (g/L)	2.3 (2.1, 2.7)	2.4 (2.1, 2.8)	−0.42	0.68
Glu (mmol/L)	4.9 (4.6, 5.2)	5.0 (4.6, 5.7)	−1.98	0.04 ^*^
TG (mmol/L)	1.4 (0.9, 1.8)	1.4 (0.9, 2.1)	−1.16	0.25
CH (mmol/L)	4.8 ± 0.1	4.9 (4.2, 5.7)	−0.84	0.39
HDL (mmol/L)	1.3 (1.0, 1.5)	1.3 (1.1,1.5)	−0.48	0.64
LDL (mmol/L)	2.9 ± 0.1	2.9 (2.3, 3.5)	−0.38	0.70
Hcy (μmol/L)	10.7 (7.9, 14.9)	9.4 (7.4, 12.2)	−2.27	0.02 ^*^

PTA, pure-tone average; NLR, neutrophil lymphocyte ratio; MLR, monocyte lymphocyte ratio; PLR, platelet lymphocyte ratio; TBIL, Total bilirubin levels; IBIL, indirect bilirubin; SOD, superoxide dismutase; Glu, glucose; TG, triglyceride; CH, cholesterol; HDL, high-density lipoprotein; LDL, low-density lipoprotein; Hcy, homocysteine.

^*^P < 0.05.

**Table 2:** Demographics and clinical variables of patients (after PSM).

**Variables**	**Case group (*n* = 47)**	**Control group (*n* = 174)**	**Statistics**	***P*-value**
Age of onset	38 (31, 53)	43.5 (31.8, 52.3)	−0.66	0.51
Males: Females	35:12	125:49	0.13	0.72
Side (Left: Right)	22:25	83:91	0.01	0.91
Time to treatment (days)	9 (4, 20)	6 (3.0, 10.3)	−3.02	0.003^*^
Hypertension	5 (10.6%)	25 (14.4%)	0.44	0.51
Diabetes mellitus	1 (2.1%)	15 (8.6%)	–	0.20
Initial PTA (dB)	61.6 ± 3.3	63.5 ± 2.2	0.43	0.67
Final PTA (dB)	50.5 ± 3.7	38.1 (22.5, 63.8)	−2.39	0.02^*^
Hearing gain (dB)	6.3 (1.3, 20)	14.4 (5, 31.6)	−3.46	0.001^*^
Tinnitus	42 (89.4%)	161 (92.5%)	–	0.55
Vertigo	16 (34%)	82 (47.1%)	2.57	0.11
Aural fulness	36 (76.6%)	114 (65.5%)	2.08	0.15
Vestibular dysfunction	12 (25.5%)	142 (81.6%)	55.1	0.00^*^
Inner ear MRI (+)	12 (25.5%)	70 (40.2%)	3.43	0.06
Thyroid dysfunction	8 (17.4%)	39 (22.4%)	0.55	0.46
Effective cases	15 (31.9%)	87 (50%)	4.87	0.03^*^
**Blood index**				
NLR	1.9 (1.4, 2.5)	1.8 (1.5, 2.5)	−0.12	0.91
MLR	0.19 (0.15, 0.29)	0.19 (0.15, 0.24)	−1.53	0.13
PLR	117.2 (88.1, 136.7)	111.5 (91.3, 145.1)	−0.89	0.37
TBIL (μmol/L)	13.1 (10.7, 15.9)	14.1 (11.3, 18.6)	−1.28	0.20
IBIL (μmol/L)	9.3 (7.4, 11.2)	10.2 (7.9, 13.2)	−1.51	0.13
SOD (U/mL)	165.7 ± 4.7	169.5 (141, 198.5)	−1.19	0.23
Fibrinogen (g/L)	2.3 (2.1, 2.7)	2.4 (2.0, 2.8)	−0.68	0.49
Glu (mmol/L)	4.9 (4.6, 5.2)	4.9 (4.5, 5.8)	−0.55	0.58
TG (mmol/L)	1.4 (0.9, 1.7)	1.4 (1.0, 1.9)	−1.15	0.25
CH (mmol/L)	4.8 ± 0.1	4.83 ± 0.08	0.33	0.74
HDL (mmol/L)	1.3 (1.0, 1.5)	1.3 ± 0.02	−0.03	0.97
LDL (mmol/L)	2.9 ± 0.1	2.9 ± 0.07	0.09	0.93
Hcy (μmol/L)	10.8 (8.0, 15.2)	10.4 (8.1, 13.5)	−0.77	0.44

PTA, pure-tone average; NLR, neutrophil lymphocyte ratio; MLR, monocyte lymphocyte ratio; PLR, platelet lymphocyte ratio; TBIL, total bilirubin levels; IBIL, indirect bilirubin; SOD, superoxide dismutase; Glu, glucose; TG, triglyceride; CH, cholesterol; HDL, high-density lipoprotein; LDL, low-density lipoprotein; Hcy, homocysteine.

^*^P < 0.05.

### 3.2. Correlations between clinical variables and treatment outcome in the case group

Univariate logistic regression analysis was performed to analyze the age, sex, time to treatment, initial PTA, final PTA, combined hypertension, the incidence of vertigo, tinnitus, aural fulness, the rate of vestibular dysfunction, inner ear MRI abnormality, and various blood parameters. The time to treatment and final PTA were significantly different between the effective and ineffective groups (95% confidence interval: 0.758–0.959 and 0.923–0.988, *P* < 0.05), as shown in Table 3. Clinical variables that yielded a *P*-value of < 0.2 in the univariate logistic regression analysis, such as time to treatment, final PTA, tinnitus, and Glu and Hcy levels, were included in the multivariate binomial logistic regression analysis. Time to treatment (95% confidence interval: 0.692–0.965, *P* < 0.05) and final PTA (95% confidence interval: 0.921–0.998, *P* < 0.05) were associated with the treatment outcomes for the case group (Table 4).

**Table 3:** Univariate logistic regression analysis for the case group.

**Variables**	**Ineffective group (*n* = 32)**	**Effective group (*n* = 15)**	**OR**	**95%CI**	***P*-value**
Age of onset	38.0 (31.0, 53.3)	33.0 (27.0,53.0)	0.990	0.948–1.034	0.657
Males: Females	25:7	10:5	1.786	0.457–6.971	0.404
Time to treatment (days)	15.0 (7.3,24.5)	4.0 (3.0,11.0)	0.853	0.758–0.959	0.008^*^
Initial PTA	61.4 ± 3.7	61.8 ± 6.8	1.001	0.973–1.029	0.955
Final PTA	57.9 ± 3.9	34.8 ± 6.6	0.954	0.923–0.988	0.007^*^
Hypertension	29 (90.6%)	13 (86.7%)	1.487	0.221–9.993	0.683
Vertigo	21 (65.6%)	10 (66.7%)	0.955	0.261–3.495	0.944
Tinnitus	2 (6.3%)	3 (20%)	0.267	0.039–1.801	0.175
Aural fullness	8 (25.0%)	3 (20%)	1.333	0.298–5.957	0.706
Vestibular dysfunction	25 (78.1%)	10 (66.7%)	1.786	0.457–6.971	0.404
Thyroid dysfunction	27 (84.4%)	12 (80%)	1.350	0.277–6.585	0.711
Inner ear MRI (+)	25 (78.1%)	10 (66.7%)	1.786	0.457–6.971	0.404
**Blood index**					
NLR	1.9 (1.4, 2.5)	1.7 (1.3,2.5)	0.844	0.528–1.351	0.481
MLR	0.19 (0.14,0.29)	0.19 (0.16,0.33)	0.581	0.064–5.250	0.629
PLR	118.3 (87.6, 140.0)	117.2 (97.2, 134.9)	1.007	0.993–1.020	0.331
TBIL (μmol/L)	13.1 (10.6, 16.3)	13.1 (10.7, 15.9)	0.965	0.847–1.100	0.597
IBIL (μmol/L)	9.2 (7.4, 11.5)	9.8 (7.7, 11.1)	0.963	0.813–1.141	0.666
SOD (U/mL)	157.5 (147.5, 193.3)	156.0 (133.0, 176.0)	0.989	0.969–1.010	0.305
Fibrinogen (g/L)	2.3 (2.1, 2.7)	2.4 (1.9, 2.8)	0.809	0.313–2.091	0.662
Glu (mmol/L)	4.9 (4.7, 5.3)	4.7 (4.1, 5.2)	0.507	0.186–1.384	0.185
TG (mmol/L)	1.3 (0.8, 1.6)	1.5 (0.9,1.8)	0.867	0.429–1.751	0.690
CH (mmol/L)	4.9 ± 0.2	4.7 ± 0.3	0.818	0.412–1.623	0.565
HDL (mmol/L)	1.3 (1.1, 1.5)	1.2 (0.9, 1.7)	0.401	0.049–3.302	0.396
LDL (mmol/L)	2.9 ± 0.1	2.9 ± 0.2	0.995	0.460–2.153	0.990
Hcy (μmol/L)	10.7 (8.3, 14.6)	11.5 (7.5, 25.1)	1.064	0.979–1.156	0.142

PTA, pure-tone average; NLR, neutrophil lymphocyte ratio; MLR, monocyte lymphocyte ratio; PLR, platelet lymphocyte ratio; TBIL, total bilirubin levels; IBIL, indirect bilirubin; SOD, superoxide dismutase; Glu, glucose; TG, triglyceride; CH, cholesterol; HDL, high-density lipoprotein; LDL, low-density lipoprotein; Hcy, homocysteine.

^*^P < 0.05.

**Table 4:** Multivariate logistic regression analysis for the case group.

	**OR**	**95%CI**	***P*-value**
Time to treatment	0.817	0.692–0.965	0.017^*^
Final PTA	0.959	0.921–0.998	0.039^*^
Tinnitus	0.270	0.026–2.804	0.273
Glu (mmol/L)	0.391	0.107–1.426	0.155
Hcy (μmol/L)	1.031	0.925–1.149	0.584

PTA, pure-tone average; Glu, glucose; Hcy, homocysteine.

^*^P < 0.05.

## 4. Discussion

The exact etiopathogenesis of SSNHL remains unknown; however, the commonly accepted etiological mechanisms for this condition include microcirculatory dysfunction of the inner ear, viral infection, and immune inflammatory states (Koçak et al., 2017; Li et al., 2018; Chen et al., 2019). Most people with SSNHL have no obvious inducing factors before the onset, but some have a variety of high-risk triggers such as noise exposure, insomnia, cold, and emotional stress. To the best of our knowledge, this study is the first to show the differences in outcomes after exposure to environmental noise triggers and the absence of obvious inducing factors before onset in patients with unilateral SSNHL. We found significant differences in the time to treatment, the audiogram curve types, the rate of vestibular dysfunction, and the effective rate between the two groups after PSM for sex, age, and initial PTA. Although a correlation between blood parameters and the prognosis was not found in the case group, the time to treatment and final PTA were positively and independently associated with the treatment outcomes of unilateral SSNHL.

Hypertension and diabetes mellitus are associated with the prognostication of SSNHL (Lin et al., 2016; Fasano et al., 2017). The onset of hypertension and diabetes is closely related to age and blood indicators, such as HDL and bilirubin level, which, in turn, are also associated with sex and age (von Mühlen et al., 2003; Gazzin et al., 2016). The degree of HL before treatment is closely related to the treatment outcomes. Therefore, to better investigate the clinical characteristics and efficacy of the case and control groups of patients with unilateral SSNHL, the effects of sex, age, initial PTA, and the associated confounding factors in this study had been excluded using PSM. Before PSM, the incidence of simultaneous diabetes mellitus, the incidence of vertigo and aural fullness, the rate of inner ear MRI abnormalities, and the Glu and Hcy levels were significantly different; however, these disparities disappeared after PSM. This may be because the PSM achieved a better balance between the confounding factors in the two groups.

Previous studies have demonstrated that vestibular dysfunction is more common in SSNHL patients with profound hearing impairment and that it correlates with hearing recovery in SSNHL (Wang et al., 2020; Chang et al., 2021). The rate of vestibular dysfunction in the case group was significantly lower than that in the control group before or after PSM. This suggests that patients with unilateral SSNHL are less likely to experience vestibular dysfunction after exposure to environmental noise triggers before onset. This may be because the location and extent of inner ear damage vary between subjects with the same degree of HL.

Different audiogram curve types may suggest the existence of different pathogeneses. Flat-type audiogram curves are mostly associated with inner ear vascular spasms or stria vascularis dysfunction; total deafness-type audiogram curves usually occur due to inner ear vessel embolism or thrombosis; sloping-type audiogram curves occur mostly due to hair cell damage; and ascending-type audiogram curves are generally associated with endolymphatic hydrops (Michel, 2011). In this study, flat-type and sloping-type audiogram curves were the most and least commonly observed types of audiogram curves, respectively, in the case group. This suggests that exposure to environmental noise triggers before onset may primarily cause inner ear vascular spasms or stria vascularis dysfunction. In conclusion, the effective rate in the case group was significantly lower than that in the control group despite the matching initial PTA. The prognostic analysis of the case group demonstrated that time to treatment and final PTA was associated with the prognosis. The mean time to treatment in the ineffective group (15 days) was longer than that in the effective group (4 days). Consequently, the treatment efficacy decreases with time. This also suggests, in part, that patients should be more vigilant after exposure to environmental noise.

Noise damage can cause the depletion of glutathione. Glutathione is an antioxidant that protects cells from damage caused by toxins, such as free radicals (Le Prell et al., 2007). High Hcy levels can affect the production of glutathione, causing further oxidative damage and microvascular ischemia (Lai and Kan, 2015). Hyperhomocysteinemia was demonstrated to serve as a poor prognostic factor for hearing recovery in SSNHL (Passamonti et al., 2015). In this study, the Hcy level in the case group was higher than that in the control group before PSM, but this difference vanished after PSM. This suggests that oxidative stress damage may not be a major etiological mechanism for the case group.

However, this study has several limitations. First, this was a retrospective study based on routine clinical records and data; therefore, some records are incomplete. Second, although most patients were exposed to environmental noise triggers before the onset of SSNHL occasionally as well as transiently, the types of noise were different, and the exposure time was not fixed. However, this study is important because, to the best of our knowledge, it is the first to analyze the differences between unilateral SSNHL caused due to exposure to noise and that with no obvious inducing factors before onset. Moreover, this study matched the relevant variables that could affect baseline characteristics in both the patient groups, such as sex, age, and initial hearing threshold; hence, it can yield highly reliable results. Nevertheless, further studies are necessary to analyze more variables and prognostic factors in a larger number of patients.

## 5. Conclusion

Patients with unilateral SSNHL with exposure to environmental noise triggers before onset mainly exhibited flat-type hearing loss and showed a poorer effective rate, longer time to treatment, and lower rate of vestibular dysfunction than those in the unilateral SSNHL patients for whom there was an absence of obvious inducing factors before onset. Time to treatment and final PTA were associated with the prognosis of unilateral SSNHL with exposure to recreational or lifestyle noise triggers before onset.

## Data availability statement

The original contributions presented in the study are included in the article/Supplementary material, further inquiries can be directed to the corresponding author.

## Ethics statement

The studies involving human participants were reviewed and approved by Shandong Provincial ENT Hospital (XYK20180102). Written informed consent for participation was not required for this study in accordance with the national legislation and the institutional requirements.

## Author contributions

YW and MW designed the study and wrote the manuscript. WX, XS, KL, and FD undertook the research and analyzed the data. HW funded the research. All authors contributed to the article and approved the submitted version.
