# Supplementary material for: Impact of environmental noise exposure as an inducing factor on the prognosis of sudden sensorineural hearing loss: a retrospective case–control study
Source: Front Neurosci. 2023 Jun 29;17:1210291. doi: 10.3389/fnins.2023.1210291 (PMC10339706; doi:10.3389/fnins.2023.1210291)
Supplement: Supplementary file 2 [file Table_2.DOCX]

The 31 cases excluded from the case group included：

4 cases > 70 years old

3 cases less than 18 years old

3 cases time to treatment was greater than 30 days

1 case of fluctuating deafness

1 case with middle ear lesions

14 cases with binaural involvement

1 case without vestibular function results

2 cases without inner ear MRI result

2 cases without complete blood test results

The 1983 cases excluded from the control group included：

118 cases > 70 years old

235 cases younger than 18 years

115 cases with bilateral ear involvement

194 cases time to treatment was greater than 30 days

10 cases of fluctuating deafness

8 cases of autoimmune disease (2 cases of rheumatic heart disease, 3 cases of ulcerative colitis and 3 cases of rheumatoid arthritis)

321 cases hospitalized for less than 7 days

12 cases of vestibular Schwannoma

12 cases of middle ear malformation

348 cases of history of previous hearing loss or contralateral deafness

80 cases of history of otitis media

9 cases of history of middle ear surgery

18 cases of history of head trauma

3 cases of Meniere's disease

334 without complete vestibular function test results

68 without inner ear MRI result

6 cases with cranial disease

89 cases without complete blood test results

3 cases without complete audiology data
